# Age-Friendly University Campus Inventory

**DOI:** 10.1093/geroni/igaa057.020

**Published:** 2020-12-16

**Authors:** Maria Claver, Adriana Weathersby, Alexandra Wilkinson, Nicole Smith

**Affiliations:** 1 California State University, Long Beach, Whittier, California, United States; 2 California State University, Long Beach, Long Beach, California, United States

## Abstract

An increasing number of older adults are turning to educational institutions to provide career training, intellectual stimulation and social interaction. Response to the educational needs and interests of this emerging population calls for new opportunities and innovative practices of teaching, research, and community engagement that colleges and universities are poised to offer. In Summer 2018, CSULB became the third university in California to be designated an Age-Friendly University. This study aimed to identify strengths and opportunities for growth at CSULB as related to each of the 10 Age-Friendly University principles. This study is part of a larger, national study conducted by researchers from UMass, Boston. Interviewees were asked to indicate on the Campus Inventory survey tool the activities and programs offered by the university. Data were collected from CSULB administrators through email after a phone introduction and entered into an Excel document for analysis using qualitative methods. Results provided a clear overview of the types of activities and programs available to older adult students and guided the identification of gaps in services, from the perspective of administrators on campus. The results of this CSULB survey will be compared with results from several AFU campuses across the United States. Additionally, results will be compared with a future Campus Climate survey, which will assess the age-friendliness of CSULB from the perspectives of faculty, students and community members.

